# A simplified computational liver perfusion model, with applications to organ preservation

**DOI:** 10.1038/s41598-025-85170-4

**Published:** 2025-01-16

**Authors:** Daniel Emerson, Yoed Rabin, Levent Burak Kara

**Affiliations:** Department of Mechanical Engineering, Carnegie Mellon University, Pittsburgh, PA 15213 USA

**Keywords:** Mechanical engineering, Gastrointestinal models, Computational science

## Abstract

Advanced liver preservation strategies could revolutionize liver transplantation by extending preservation time, thereby allowing for broader availability and better matching of transplants. However, developing new cryopreservation protocols requires exploration of a complex design space, further complicated by the scarcity of real human livers to experiment upon. We aim to create computational models of the liver to aid in the development of new cryopreservation protocols. Towards this goal, we present an approach for generating 3D models of the liver vasculature by building upon the space colonization algorithm. Additionally, we introduce the concept of a super lobule which enables a computational abstraction of biological liver lobules. User-tunable parameters allow for vasculatures of varying depth and topology to be generated. In each model, we solve for a common lumped resistance value assigned to the super lobules, allowing the overall physiological blood pressure and flow rate through the liver to be preserved. We demonstrate our approach’s ability to maintain consistency between models of varying depth. Finally, we simulate steady state machine perfusion of the generated models and demonstrate how they can be used to quickly test the effect of different boundary conditions when designing organ preservation protocols.

## Introduction

Liver transplantation is the primary treatment option for people with end-stage liver disease^1^. However, one major barrier to the increased prevalence of liver transplantation is the absence of advanced methods for liver preservation. The current standard in preservation, static cold storage (SCS), limits preservation of human livers to less than 12 hours. In SCS the liver is simply flushed and immersed in a preservation solution at 4 °C, and transported on ice. Researchers have shown that as the liver is cooled to lower temperatures, the metabolic rate decreases and consequentially the rate of deterioration decreases^2^. Advanced preservation techniques known as subzero preservation or supercooling aim too cool the liver below the SCS standard, allowing us to extend the life of transplantable livers beyond current limitations^3^. However, development of advanced preservation methods is a costly and time consuming process. First, it is exceedingly difficult to obtain real human livers to conduct experiments, with researchers more often relying on rat and pig livers, or partial liver grafts for preliminary experiments. Furthermore, successful preservation protocols are highly complex processes involving cooling and reheating of the organ at different temperatures, all while loading and unloading the organ with cryoprotective agents (CPAs)^2–6^. While loading and unloading the organ, clinicians have minimal control over where the CPA travels and are usually limited to controlling the time-dependent pressure and flow rate at which it is loaded. A better understanding of the rate, location, and concentration of CPA throughout the vasculature could help clinicians and researchers better understand localized issues that can damage the organ during preservation. Additionally, there are many challenges to be addressed during cryopreservation including: ischemic damage, ice crystallization, CPA toxicity, structural damage due to thermal stress, and the accumulation of waste products^7^.

As such, it could be cost-effective and time-efficient to conduct the early phase of preservation study on computational models of the liver. In particular, our long term objective is to develop appropriate computational models, wherein the fluid flow in the liver can be modeled and simulated, allowing end users to virtually experiment with different CPA loading protocols, optimize process variables, and ultimately benchmark its outcomes against preservation protocols applied to real livers. However, the highly complex anatomical structure of the liver, coupled with its intricate vasculature network and lobules, makes it challenging to produce computational models suitable to CPA flow simulations.There are roughly one million lobules in the liver, which makes it very challenging to create a model which captures the true complexity of the human liver. These lobules are often abstracted as tessellated hexagons, with the portal arterioles and portal venules located at the vertices of the hexagon, connecting to the central vein through sinusoids^8^. To date, there have been two primary approaches to creating models of the liver vasculature: anatomical and algorithmic approaches.

Researchers have created anatomical models of the human liver vasculature using computed tomography (CT) scans^9,10^, and with vascular corrosion casting (VCC)^11,12^. Anatomical models have the advantage of being based on a real human liver vasculature, but are costly and time prohibitive to generate, yielding only a single model. The depth of the model is limited based on the CT imaging resolution, or casting resin perfusion depth^12^. Previously generated anatomical models do not fully connect the inlet networks (PV and HA) to the outlet network (HV). Instead, the anatomical models are used to aggregate statistics about the vasculature such as vessel counts, average diameter and length^8,11,12^, which can be used to generated low dimensional lumped parameter models for simulating flow through the entire vasculature^11,13^. Anatomical models have also been used in detailed flow simulations, but only for a single network at a time, with only major vessels^10^. Towards our goal of understanding how CPA flows through the liver vasculature, we require the inlet and outlet vasculatures to be fully connected, and we need to retain the 3D spatial information of all of the vessels in our model.

Karch et al. developed the constrained constructive optimization (CCO) algorithm to generate arterial trees^14^. The CCO algorithm generates the vascular tree by iteratively adding new vessels and solving a biologically inspired cost function. When generating new vessels, the algorithm assigns radii according to the Hagen-Poiseulle equation. Schwen and Preusser use CCO to generate models of the portal vein, hepatic vein and hepatic artery, and compare the generated models with real livers, but do not connect the models or simulate flow^15^. Guy et al. made improvements to the CCO algorithm to reduce model generation time^16^. Talou et al. and Jessen et al. build on the CCO algorithm to generate vascular models which closely agree with anatomical model data^17,18^. While the CCO algorithm quickly generates realistic models of the liver vasculature, existing publications have not created a fully connected model that simulates flow throughout the entire vasculature of the liver in three dimensions. This is in part due to the fact that the CCO algorithm does not provide explicit control over the trajectory or terminal location of the generated vessels. Therefore, there is not a natural mechanism to generate a model which is connected at the terminals of the inlet and outlet networks. Guy et al. generate connected networks by modifying previously generated vessels, but this approach negates any previous optimization done, leading to unrealistic looking vascular structures^16^.

Between purely anatomical and purely algorithmic models, there have been several 0D and 1D models created based on the anatomy of real livers which simulate flow with an electrical-fluid flow analogy. Debbaut et al. simulate hypothermic machine perfusion of a lumped parameter model which is based on anatomical corrosion cast and imaging data^11^. Wang et al. extends the lumped parameter model from Debbaut et al. to represent the Couinaud segmentation of the liver and investigate pressure gradients in cirrhotic livers. Lastly, Tithof et al. create a model which simulates flow through up to millions of vessels for analyzing flow alterations due to partial hepatectomies^13^. While Wang et al. and Tithof et al. retain some spatial information from the vessels, we require our model retain full three-dimensional spatial relationships between vessels because we plan to conduct heat transfer simulations in future works. That said, we solve the fluid flow through our model in a manner similar to Tithof et al., and would like to include effect of capacitances like Debbaut et al. and Wang et al. in future works.

**Fig. 1:**
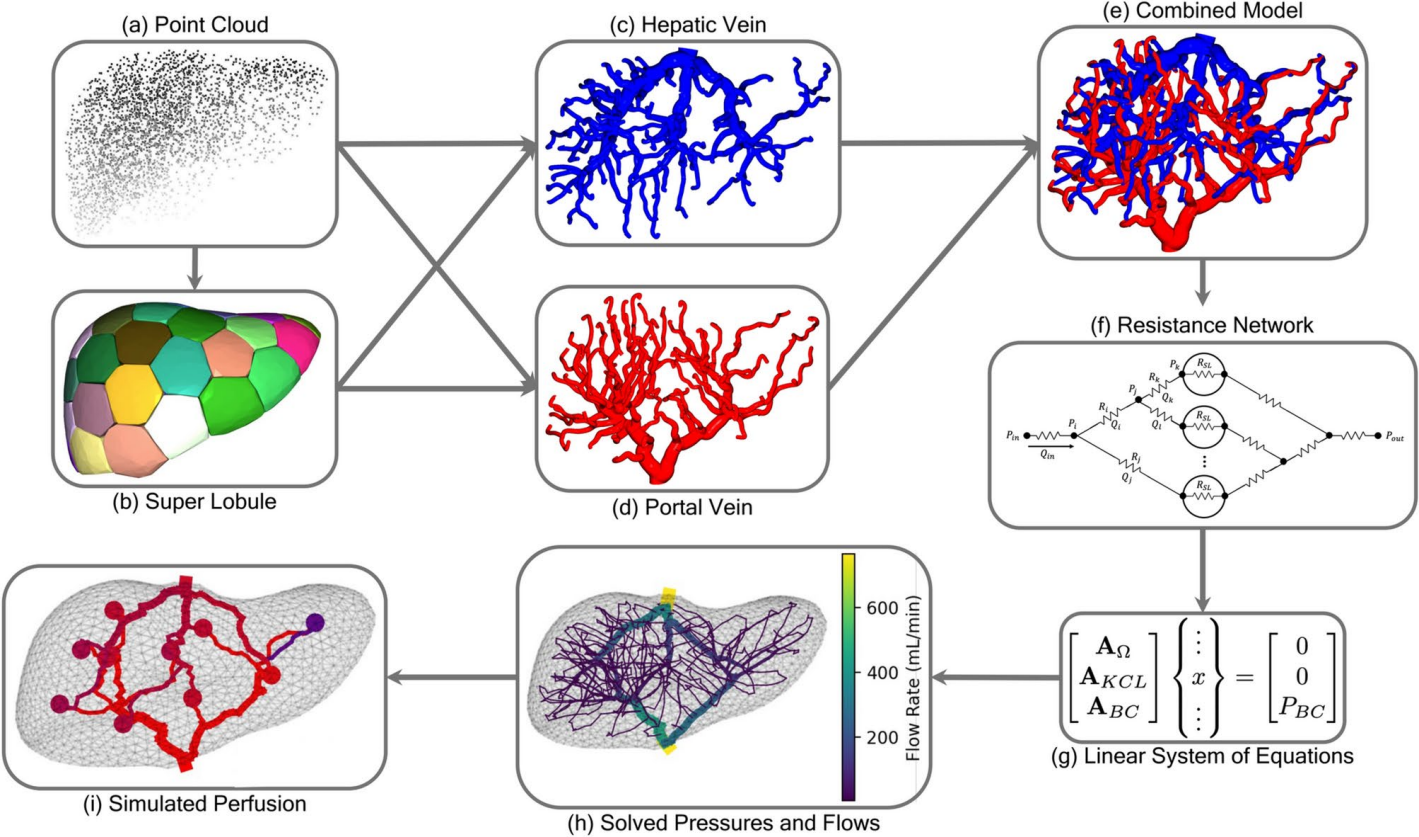
Overview of the vascular generation in the liver and subsequent simulated perfusion. (**a**) We begin with a volumetric point cloud uniformly sampled within the volume of a liver model to be vascularized. (**b**) Using k-means, we partition the liver volume into abstract computational constructs we call super lobules. (**c**) Using the space colonization algorithm, the vasculature for the hepatic vein is generated until all super lobules are reached. (**d**) A similar process is repeated for the portal vein vasculature. (**e**) The two vasculatures are connected at the super lobules to create a fully connected model. (**f**) Using the lengths and radii of the vessels in the model, a resistance network is constructed. (**g**) Circuit laws are used to create a linear system of equations. (**h**) The system is solved to find the pressures and flow rates throughout the model. (**i**) This approach can then be used to simulate steady state perfusion of the vessels.

In this paper, we describe a new approach for generating three-dimensional algorithmic models of the human liver vasculature and its utility in a simplified CPA perfusion simulation. Figure 1 summarizes our approach. Our approach is inspired by the growth of botanical trees and builds upon the space colonization algorithm first introduced by Runions et al.^19,20^. Utilizing additions to the algorithm from Ulu and Kara we are able to generate vascular models that are guaranteed to reach specified terminal locations^21^. By enforcing the location and number of terminal vessels, we connect the inlet and outlet vascular trees to create a fully connected vascular network where flow can be simulated through the entire model. Model depth is user-controllable, and is varied by changing the number of terminal vessels. Model topology is varied by changing parameters in the space colonization algorithm.

As a key component underyling our approach, we introduce a new concept we call a ‘super lobule’. A super lobule is computational construct that aims to represent a spatially proximate group of biological lobules found in the liver. Similar to its biological counterpart, a super lobule serves as one of many connection points between the inlet and outlet vascular trees. A super lobule models an arbitrary grouping of hepatocyte cells, arterioles, venules, and sinusoidal vessels, all modeled with a single lumped resistance. A key utility of this abstraction is that the number of super lobules is a user-controllable parameter, allowing end users to generate a wide range of models at different levels of detail. Similar to mesh convergence, we intend users to be able to increase the detail of the model by increasing the number of super lobules, until parameters of interest asymptotically converge.

Together, the space colonization algorithm and super lobule concept allow for the generation of many unique models that all meet the same pressure and flow rate boundary conditions, regardless of the number and size of the explicitly modeled vessels. We demonstrate consistency across models with various levels of detail, as well as the ability to generate models with large degrees of topological variability.

Due to the three dimensional nature of our vascular model, we are able simulate the flow of CPA and blood through the vasculature and identify difficult to perfuse regions. In this work, we use a purely resistive network that models the resistance in the vasculature as well as the suber lobules. We utilize the Hagen-Poiseuille fluid model, under the assumption that flow is incompressible, laminar, Newtonian, and occurs in long cylindrical pipes of constant cross sectional area. While there are limitations of these assumptions, they are commonly used in literature when modeling flow through vascular structures^11,13,15–18,22^. We demonstrate the use of the resulting computational model across varying flow boundary conditions as well as varying model parameters. As will be discussed, the hyperparameters in our approach allow end users to generate models at varying levels of detail, ranging from simple to complex models. We show how simulations with our model can be qualitatively compared with experimental perfusion of real human livers.

Our main contributions are:

- adaptation of the space colonization algorithm for generating fully connected vascular structures,
- introduction of the novel super lobule concept to generate models of varying depth that adhere to prescribed boundary conditions,
- simulation of flow through a 3D liver vasculature from a preservation protocol development perspective.

## Methods

We begin by introducing the details behind super lobules and how they are instantiated within a liver model. Next, we describe how the liver vasculature and the super lobules are collectively modeled as a resistive flow network. We then, describe the space colonization algorithm that underlies the vasculature generation mechanism.

### Super lobule

At a microscopic level, the liver consists of approximately one million highly regular functional units called lobules^8,23^. Each lobule is typically represented by a hexagonal prism, surrounded by six portal triads. A portal triad consists of the portal vein, hepatic artery, and bile duct. Fluid flows from the portal triad through many sinusoidal vessels and drains into the hepatic vein, sometimes called the central vein, in the middle of the lobule^8^.

To develop a computational model appropriate for preservation-related fluid flow within the liver, we propose a spatial grouping of proximate lobules in the form of an abstract construct we call a super lobule (SL). A super lobule aims to model a collection of lobules, arterioles, venules and small scale sinusoidal vessels, in the form of a single lumped unit. As will be described, given a target level of vasculature detail prescribed by the end user, each super lobule serves as a means to account for the volume and flow resistance of the microscale vasculature that is expected to exist in the corresponding volumetric region in the real liver. We note that in this work, a super lobule does not intend to model the precise metabolic activity at each biological lobule. Instead, each super lobule is designed to encode the total volume it claims within the liver, together with a resistance to blood/CPA flow it will exert while guaranteeing that computationally imposed boundary conditions are always met regardless of the level of model detail. This choice enables the development of a vasculature network amenable to organ preservation simulations such as a CPA loading of the liver.

**Fig. 2:**
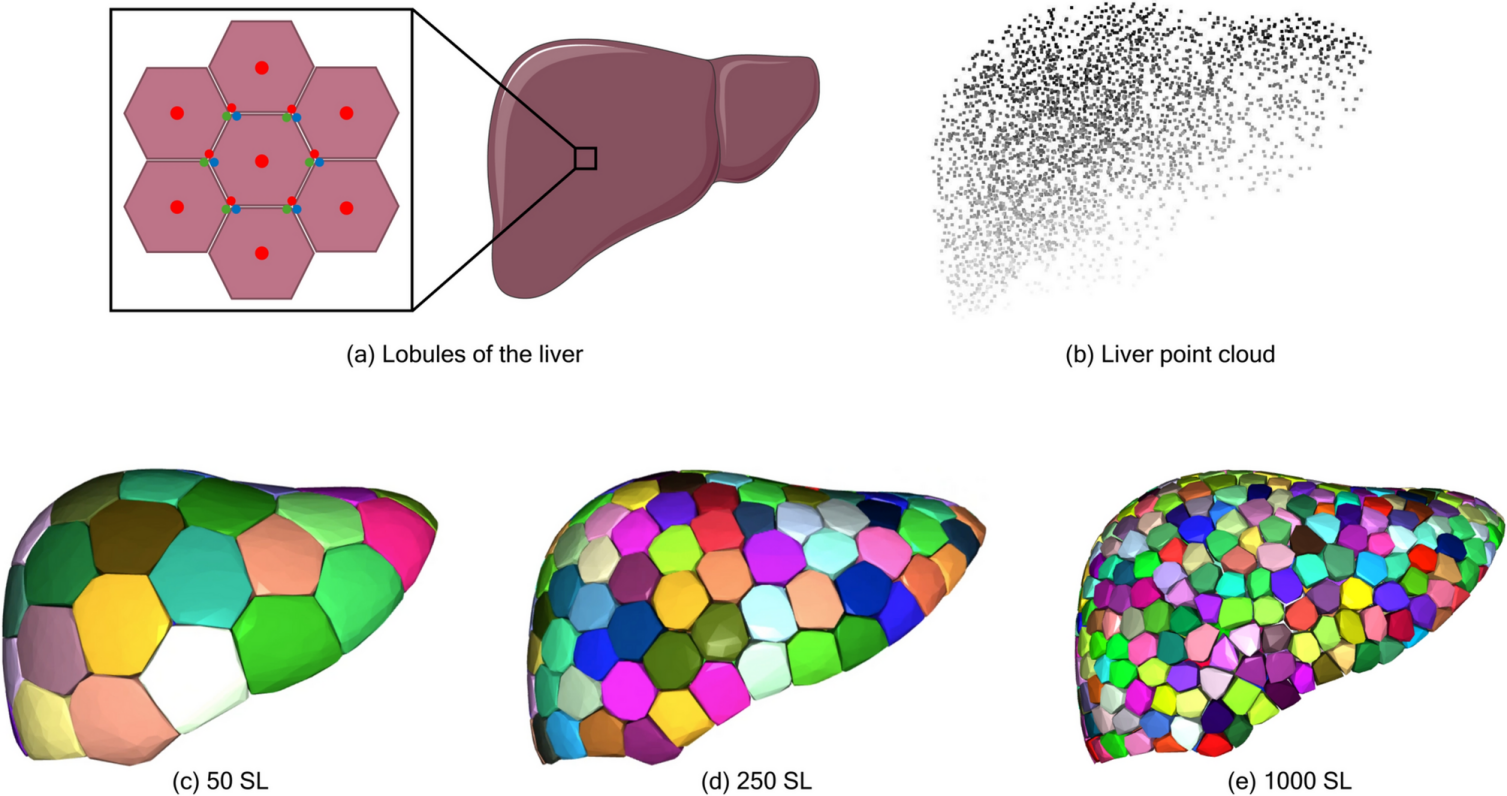
Lobules are the functional building blocks of the liver, as detailed in (**a**) [adapted from^8,24^]. We introduce the super lobule as a lumped model to account for the lobules and small scale sinusoidal and capillary vessels which we do not explicitly model. Super lobules are generated using the k-means algorithm to cluster points which are uniformly sampled within the liver volume (**b**). The number of super lobules is a user defined parameter which corresponds to models of increasing detail, as the number of super lobules increases. We visualize models with $k=50$ super lobules (**c**), $k=250$ super lobules (**d**) and $k=1000$ super lobules (**e**).

Users determine the level of detail they desire by explicitly controlling the number of super lobules. A limited number of super lobules produces simple vasculatures and eventual flow models, whereas a large number of super lobules produce a more detailed model. Ultimately, the number of super lobules represents the level of detail of the produced model, conceptually analogous to the number of elements in a finite element mesh structure. Given the number of desired super lobules as input, to locate the super lobules, we uniformly sample points inside the liver volume, and then apply k-means clustering to generate *k* super lobules. The points are sampled with a standard uniform distribution within a Cartesian bounding box that encapsulates the liver volume. Points which lie outside of the liver volume are discarded. Figure 2 shows a visualization of the initial point cloud and the convex hull corresponding to each super lobule generated with k-means.

Our choice to generate the super lobules with k-means clustering on a uniform point cloud ensures that we generate super lobules of roughly equal volume, since the k-means algorithm minimizes the sum of squared Euclidean distances for each cluster. Furthermore, since super lobules are simply comprised of lobules and small scale vessels, if we assume all lobules are of roughly equal volume, they are also of roughly equal resistance. Thus, we assume all super lobules in the model to have identical resistance and identical volume. We compute the volume assigned to the super lobules according to Equations 1 and 2.

1 $$\begin{aligned} V_{\rm SL,total}&= V_{\rm blood} - V_{\rm vessels} \end{aligned}$$

2 $$\begin{aligned} V_{\rm SL,individual}&= V_{\rm SL,total}/n_{\rm SL} \end{aligned}$$

Equation 1 computes total volume accounted for by the super lobules, $V_{\rm SL,total}$, as the difference of the total blood volume in the system, $V_{\rm blood}$, and the volume of the modeled vessels, $V_{\rm vessels}$. Equation 2 divides the total super lobule volume, $V_{\rm SL,total}$, by the number of super lobules, $n_{\rm SL}$, to determine the volume of each individual super lobule, $V_{\rm SL,individual}$. In this sense, the super lobules account for all of the blood carrying vessels and tissue in the system that are not explicitly modeled.

### Vascular resistance network

#### Modeled vessels

The liver has two sources of blood, the portal vein and the hepatic artery. The portal vein delivers nutrient rich deoxygenated blood, while the hepatic artery delivers oxygen rich blood^8^. The portal vein provides approximately 75% of the blood supply to the liver, with the remaining 25% coming from the hepatic artery^25,26^. The trajectory of the portal vein and hepatic artery closely follow each other all the way to the lobule level^8^. Furthermore, some perfusion techniques perfuse the portal vein only^27^. For these reasons, when developing this new model we decided to only model the portal vein on the inlet to reduce the complexity of our model. We then connect the portal vein to the outlet vasculature, the hepatic vein, at the super lobules.

#### Hagen-Poiseuille fluid model

We model the resistance of each blood vessel according to the Hagen-Poiseuille equation (Eq. 3) which simplifies Navier-Stokes under the assumption that the flow is incompressible, Newtonian, and laminar in a long cylindrical pipe with a constant cross sectional profile. The Hagen-Poiseuille model is commonly used in simplified models of vascular flows^11,13,15–18^. $\mu$ represents the dynamic viscosity of the fluid, *L* and *r* are the length and radius of the vessel respectively.

3 $$\begin{aligned} R = \frac{8 \mu L}{\pi r^4} \end{aligned}$$

#### Radius assignment

To assign radii to the vessels in our model we use Murray’s law as detailed in Eq. 4. Murray’s law relates the radius of a larger parent vessel, $r_{\rm parent}$, and its two child vessels $r_{{\rm child},1}$ and $r_{{\rm child},2}$, as detailed in Eq. 4, where $\gamma$ is the power law coefficient.

4 $$\begin{aligned} r_{\rm parent}^{\gamma } = r_{{\rm child},1}^{\gamma } + r_{{\rm child},2}^{\gamma } \end{aligned}$$

Murray’s law presumes that biological systems have evolved to minimize resistance to flow. For incompressible, Newtonian, and laminar flow, the value of $\gamma = 3$ can be derived from the Hagen-Poiseuille equation as detailed in^28^. Murray’s law is the standard method for assigning radii in branching vascular systems^14–18^. We can also write Murray’s law in a more abstract way to handle any number of child vessels, *C*, as shown in Eq. 5.

5 $$\begin{aligned} r_{\rm parent}^\gamma = \sum _{i = 1}^{C} r_{{\rm child},i}^\gamma \end{aligned}$$

In our case, we assign the radius of the first vessel in the vasculature, and we would like all subsequent vessel radii to be computed based on the topology of the vasculature. To achieve this, we need to restructure Murray’s law to compute the radii of all child vessels given a parent vessel radius. At any given branching point, we consider that a given child vessel may supply more vessels and subsequently more super lobules than it’s neighboring child vessel. Physiologically, we would expect the first child vessel to have a larger radius to supply more fluid the additional vessels and super lobules. Therefore we introduce $\omega$, which is a ratio of the number of super lobules downstream from a given child vessel, over the number of super lobules downstream from the parent branch. Our modified formulation is presented in Eq. 6. We note that for a parent branch with *C* number of child vessels, the sum of $\omega _i$ values equals 1 as shown in Eq. 7, illustrating that $\omega$ serves as a partition of unity, thereby obeying Murray’s law.

6 $$\begin{aligned} r_{{\rm child}, i} = \root \gamma \of {\omega _i \cdot r_{\rm parent}^\gamma } \end{aligned}$$

7 $$\begin{aligned} \sum _{i=1}^C \omega _i = 1 \end{aligned}$$

#### Electrical - fluid flow analogy

**Fig. 3:**
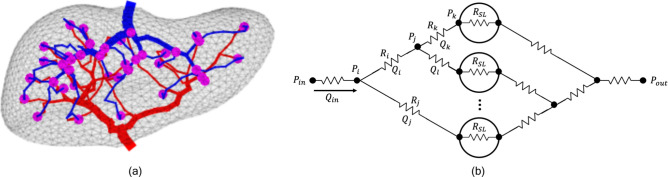
(**a**) The 3D vascular network is visualized with the portal vein (red), hepatic vein (blue) and super lobules (magenta). (**b**) The model can be converted into a 0D resistance network using the lengths, radii, and connectivity of the vessels where each vessel has a resistance and a flow rate (current) and each node has a pressure (voltage).

To simulate flow through our vascular model, we consider a simple electric analog model where voltage and current are analogous to pressure and flow rate^29^. The portal vein and hepatic vein networks are generated with the space colonization algorithm and are connected at corresponding super lobules to create one fully connected system as shown in Fig. 3a. The 3D vascular network is then used to create a 0D resistance network with resistances representing all vessels and super lobules in the model. After prescribing pressure boundary conditions at the inlet and outlet, the pressures and flows can be solved at every node and vessel in the model respectively.

#### Construction of linear system of equations

We construct a linear system of equations corresponding to the 0D resistance network of the form ${\textbf{A}} \cdot x = b$ based on the generated network’s resistances as shown in Eq. 8 for the network in Fig. 3b. The flow rates across the model’s *j* vessels, *k* super lobules and pressures at the *l* nodes are represented by $x \in {\mathbb {R}}^{n \times 1}$ where $n = j+k+l$. There are three equations used to construct the coefficient matrix in Eq. 8. First, we consider Ohm’s law ($\Delta P - QR = 0$); the pressure drop across all vessels and super lobules as ${\textbf{A}}_{\Omega } \in {\mathbb {R}}^{(j+k) \times n}$. Next, we consider Kirchhoff’s current law ($\sum Q_{in} - \sum Q_{out} = 0$) at all nodes except the two boundary nodes, as ${\textbf{A}}_{KCL} \in {\mathbb {R}}^{(l-2) \times n}$. Finally, we enforce the boundary conditions as constant pressures at the inlet and outlet as ${\textbf{A}}_{BC} \in {\mathbb {R}}^{2 \times n}$. The flows and pressures in *x* are solved using an LU decomposition of the dense asymmetric coefficient matrix ${\textbf{A}}$.

8 $$\begin{aligned} \begin{bmatrix} {\textbf{A}}_{\Omega } \\ {\textbf{A}}_{KCL} \\ {\textbf{A}}_{BC} \end{bmatrix} \begin{Bmatrix} \vdots \\ x \\ \vdots \end{Bmatrix} = \begin{bmatrix} 0 \\ 0 \\ P_{BC} \end{bmatrix} \end{aligned}$$

#### Root finding super lobule resistance

While we can compute the resistance of a vessel based on its geometry, we do not have a deterministic way to compute the lumped resistance of the super lobules $R_{SL}$. Instead, we prescribe a flow rate boundary condition on the system, and then solve for the lumped resistance of the super lobules, $R_{SL}$, which satisfies the pressure and flow rate boundary conditions. We use the bisection root finding method to query the network in Eq. 8 with several values of $R_{SL}$ and fixed pressure boundary conditions until our solution converges to the desired model flow rate. By solving for the super lobule lumped resistance we can prescribe models of varying levels of detail and topology to have the same bulk flow characteristics.

### Space colonization algorithm

To generate the vascular structure of the liver, we develop upon the space colonization algorithm introduced by Runions et al.^19,20^. The space colonization algorithm was originally inspired by the growth of botanical leaves and trees. The algorithm is an attractor based growth method, which means that the vasculature is generated by adding new vessels (growth) towards points (attractors) in the domain which have yet to be grown towards. The concept of the target attractor introduced by Ulu and Kara^21^ guarantees that the generated tree reaches specified point(s). With this, we can generate two separate trees which correspond to the portal vein and hepatic vein, and then connect the two trees at a common location such that we can simulate flow through the entire liver.

**Fig. 4:**
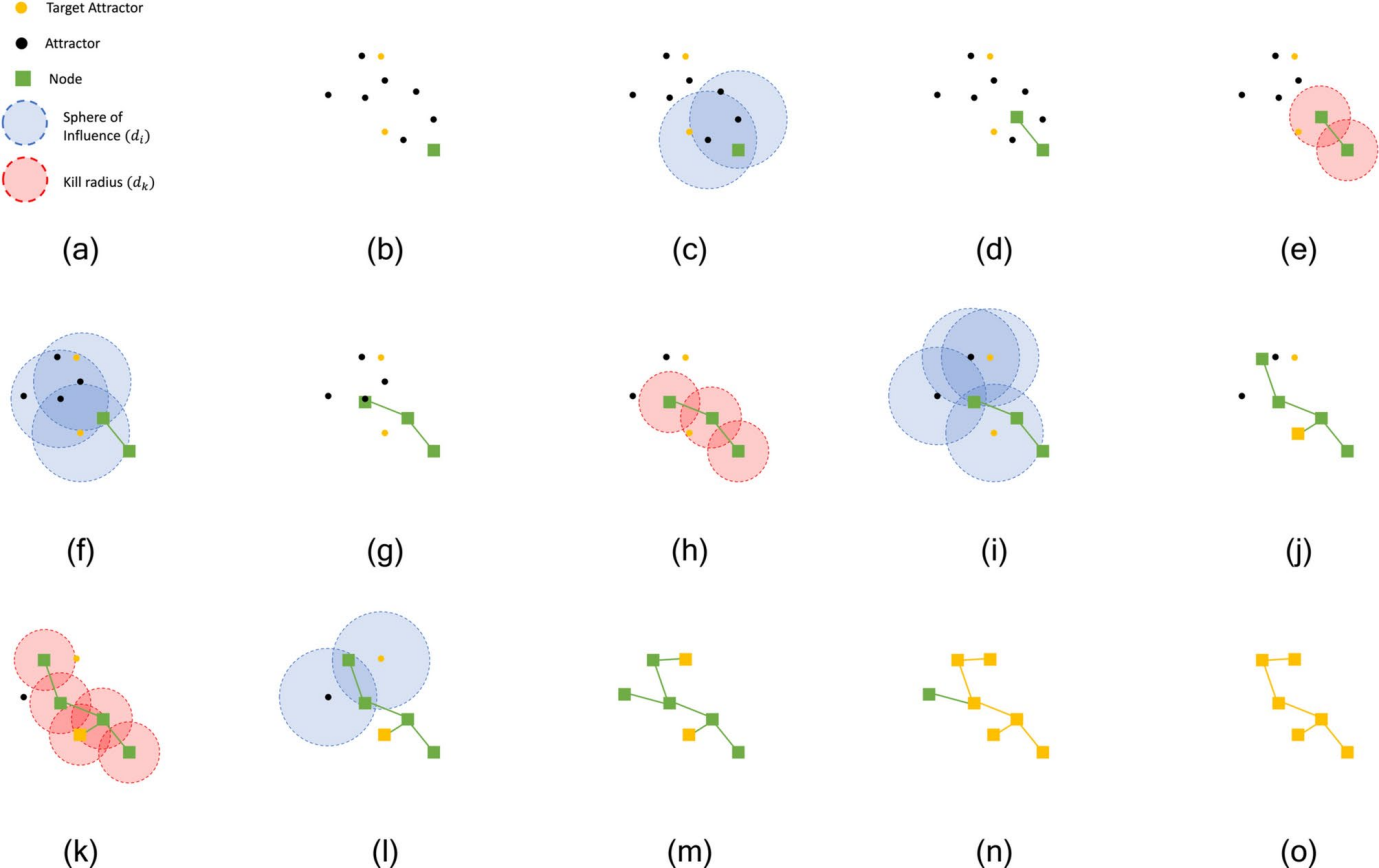
Illustration of space colonization algorithm. A key for the symbols is showing in (**a**). The point cloud is initialized with a root node, attractors, and target attractors in (**b**). At the start of each iteration we search for attractors with nodes within the sphere of influence, $d_i$ as shown in (**c, f, i, l**). We consider the attractors with nodes within $d_i$ to be “active attractors.” The nodes with active attractors then grow a new node in the average normal direction of all of it’s active attractors, at a step distance of *D,* as shown in (**d, g, j, m**). At the end of each iterations, we eliminate attractors within a kill radius $d_k$ of the existing nodes, as shown in (**e, h, k**). It is important to note that target attractors cannot be eliminated until they are explicitly reached by the tree. Once all target attractors are reached, as shown in (**m**), we traverse the tree in reverse from target attractors to the root nodes, to identify all branches critical to the tree, as shown in (**n**). Branches which are noncritical are pruned, resulting in the final tree as shown in (**o**).

#### Space colonization schematic

A schematic of the modified space colonization algorithm can be seen in Fig. 4. The algorithm begins with a specified root node and a set of attractor points. We can use the same point cloud that is generated previously, but it is important to note that the density of the attractor point cloud has a large influence on both the speed which the algorithm takes to generate a model and also the topology of the model. The centroid of the generated super lobules are specified as target attractors. With each iteration of the algorithm, nodes that are within the sphere of influence $d_i$ of any attractors grow a new node towards the average normal of the attractors at a step distance of *D*. After all nodes have been added for a given iteration, attractors that are within the kill distance $d_k$ of any node in the model are removed from the the attractor set, with the exception that target attractors cannot be removed until they are explicitly reached. When the only active attractor for a given node is a target attractor and it is within the step distance *D*, a branch is added directly to the target attractor. In this way, the generated tree grows towards regions rich with attractors, where there are no existing nodes or vessels. The algorithm terminates when all target attractors have been reached. After the tree has been generated, noncritical branches are trimmed by traversing the tree in reverse from the super lobules to the root node. Any vessels that are not traversed are removed, or pruned from the tree.

#### Intuition behind space colonization parameters

The three space colonization parameters, $D, d_k,\; {\text{and}}\; d_i$ provide the most natural way to generate models with unique topologies. The step distance parameter, *D*, determines how far new nodes are placed from the existing node from which they are grown. Large *D* values will lead to straighter, more direct looking models. The kill distance parameter, $d_k$, coupled with the sphere of influence distance, $d_i$, effectively determines the scope of attractors which are considered and subsequently removed when generating new nodes. Larger $d_i \; {\text {and}}\; d_k$ values will also lead to models that appear more direct, since they allow for more points to be considered when determining the attractor normal direction to generate new nodes in. Overall, the effect of $D, d_k, {\text{and}}\; d_i$ can generally be described that well behaved systems with $D< d_k < d_i$ illustrate the trend that for smaller values, models have a higher degree of tortuosity and for larger values, models appear more direct with straight vessels.

### Simulated loading of the vasculature

Using our completed model, we can visualize how fluid perfuses through the vasculature. We take the vessels to first be full of blood, and then load CPA at a constant flow rate, thereby displacing the blood. At $t = 0$, the concentration of a vessel, $C_t$, is initialized as $C_t = 0$, where a concentration of 0 corresponds to all blood, and a concentration of 1 corresponds to all CPA. At each time step of the simulation, the inlet flow rate is multiplied by the simulation time step to determine the volume of CPA added to the inlet vessel. Then, within a given time step, all other vessels in the model have their concentrations updated, once their parent vessel(s) have had their concentration updated. The concentration, $C_{in}$, of the incoming fluid is a weighted combination of the incoming parent vessels’ concentrations, $C_i$, proportional to their flow rates as detailed in Eq. 9. The volume of fluid added to the vessel is simply the sum of parent vessel flow rates, multiplied by the time step, *dt* as detailed in Eq. 10. Then we are able to compute the concentration of the vessel at the next time step, $C_{t+1}$ according to Eq. 11. The incoming fluid concentration is weighted by the volume of incoming fluid, and is added to the existing fluid concentration, weighted by the vessel volume, less the displaced fluid. Finally the new concentration is determined by dividing this weighted combination by the vessel volume.

9 $$\begin{aligned}&C_{in} = \frac{\sum _{i=1}^m C_i \cdot Q_i}{\sum _{i=1}^m Q_i} \end{aligned}$$

10 $$\begin{aligned}&V_{in} = \left( \sum _{i=1}^m Q_i\right) \cdot dt \end{aligned}$$

11 $$\begin{aligned}&C_{t+1} = \frac{V_{in} \cdot C_{in} + (V_{vessel} - V_{in})\cdot C_t}{V_{vessel}} \end{aligned}$$

## Results

In this section we generate various models of the liver vasculature, highlighting the self consistency, variability, and utility of the proposed methods. Our models are generated within a 1.6 L human liver volume which Pomposelli et al. found to be the average volume across 375 healthy potential liver donors^30^. The STL model of the liver is taken from Thingiverse, and smoothed and scaled to a volume of 1.6 L^31^. The bulk flow properties for CPA loading are based upon *ex vivo* subnormothermic machine perfusion (SNMP) experiments from Bruinsma et al.^32^. We choose these properties because during advanced preservation strategies, unique CPA cocktails are often loaded and unloaded in the SNMP regime as the liver is cooled or rewarmed^2–6^. The portal vein (PV) inlet pressure is set to 5.8 mmHg while the hepatic vein (HV) outlet pressure is left at 0 mmHg. The steady flow rate at the inlet is 767 mL/min. As a reminder, we do not model the hepatic artery and we simply use the pressure and flow rates for the PV from the study. The initial diameter for both PV and HV is set to $15\,{\text {mm}}$. The viscosity of blood depends on shear rate as well as the hematocrit, but since we assume Newtonian fluid flow we fix the dynamic viscosity of blood at 3.5 cP^33^. We make an additional simplification and presume the viscosity of the CPA we load to be $3.5\,{\text{cP}}$, such that there is no additional complexity due to the interface or mixing of blood and CPA. $R_{SL}$ is found using the bisection finding method, stopping when the inlet flow rate is within a tolerance of $1e-8$ of the desired flow rate with stable convergence for initial bounds ranging from $\{0,1\}$ to $\{0,10^{20}\}$. Models are generated and simulated on a 2.9GHz 12-core Intel Core i9-7920X processor.

**Fig. 5:**
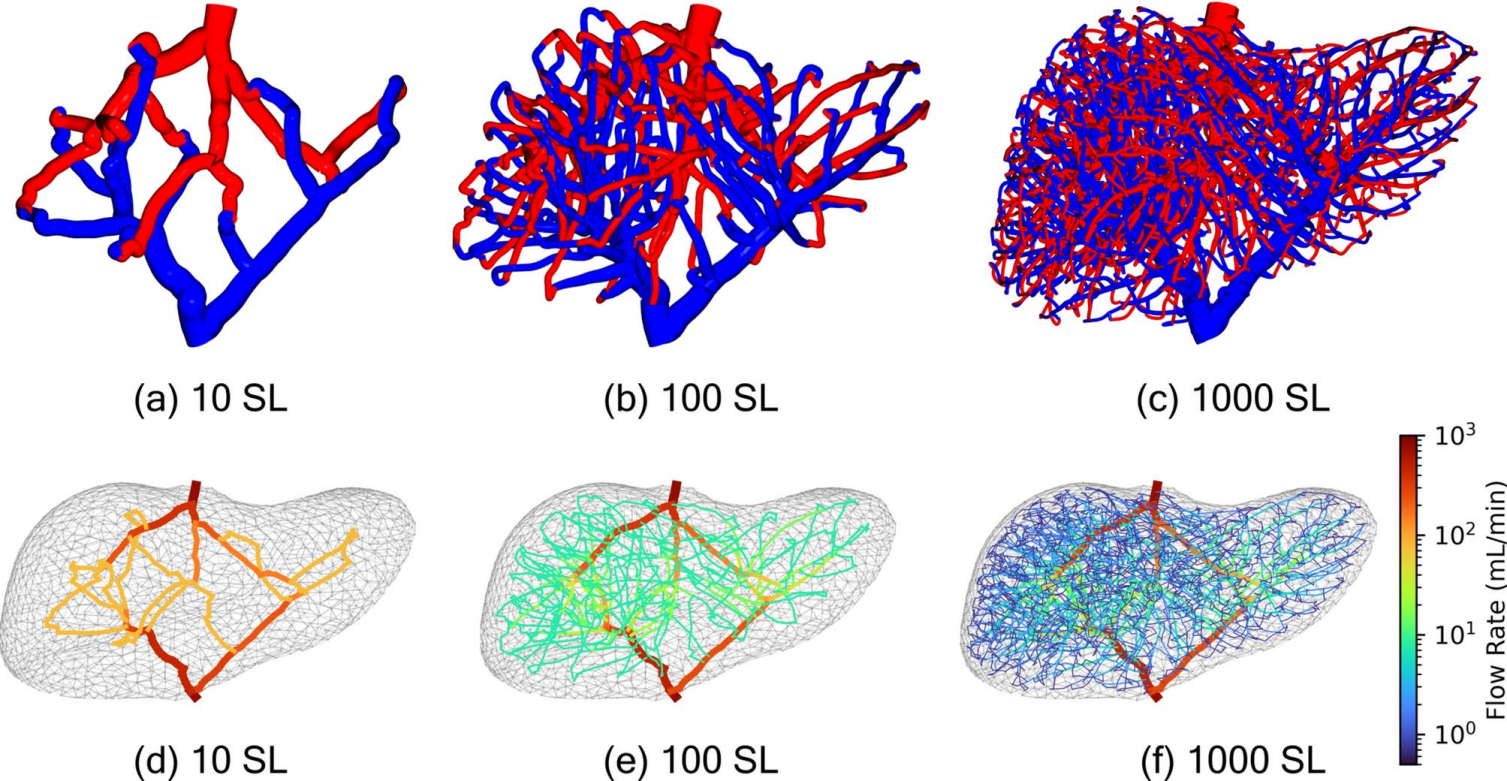
3D model visualizations and vessel flow rates for 10 (**a,d**), 100 (**b,e**), and 1000 (**c,f**) SL models with $D = 5~{\text {mm}}$, $d_k = 10~{\text {mm}}$, $d_i = 15~{\text {mm}}$.

### Model consistency

One advantage to generating models of the liver vasculature algorithmically is that we can generate many unique models by varying the algorithmic parameters. Since we constrain all of our models to meet specified boundary conditions, namely inlet and outlet pressures, and an inlet flow rate, we must determine another way to evaluate their performance. In Fig. 5 we visualize the flow rate in all vessels in models of 10, 100 and 1000 super lobules. We observe for a fixed set of space colonization parameters, $D = 5~{\text {mm}}$, $d_k = 10~{\text {mm}}$, and $d_i = 15~{\text {mm}}$, regardless of the number of super lobules in our models, the overall topology and division of flow between the models is highly similar. This is due to several reasons; firstly, in all models we initialize the left and right portal veins on the inlet tree, and the left, middle, and right hepatic veins on the outlet. These initialized vessels help ensure that the major blood vessel branches in our models are similar to those in biological livers, and also ensures that our models are self similar with each other. We note that the right portal vein sees a greater flow rate as it services a larger volume of tissue, or super lobules in our case. After initialization, the space colonization algorithm is allowed to freely determine all remaining vessels in the model until all super lobules are reached. Due to the attractor based nature of the space colonization algorithm and the uniform placement of super lobules with the k-means algorithm, all models are effectively incentivized to cover the interior domain of the liver as evenly possible, similar to the highly regular lobule structure in biological livers^8^.

**Table 1:** Properties of models with varying numbers of super lobules.

nSL	# of Vessels	Time SC (s)	Matrix size, N	Time matrix solve (s)	$R_{SL} \left( \frac{{\text {dyn-s}}}{{\text {mm}}^5}\right)$	$R_{SL,eq} \left( \frac{{\text {dyn-s}}}{{\text {mm}}^5}\right)$	$\Delta P_{SL}$ (mmHg)	$Q_{SL} \left( \frac{{\text {mL}}}{\min }\right)$
5	18	61.647	205	0.001	0.030	5.942e-03	5.698	153.400
10	38	63.828	338	0.008	0.059	5.928e-03	5.684	76.700
25	95	64.370	709	0.164	0.148	5.903e-03	5.660	30.680
50	189	66.576	1245	0.239	0.294	5.887e-03	5.645	15.340
100	375	66.982	2196	0.340	0.587	5.867e-03	5.625	7.670
250	945	66.727	4308	0.181	1.461	5.846e-03	5.605	3.068
500	1891	70.362	7126	0.560	2.915	5.829e-03	5.589	1.534
1000	3758	83.698	11676	2.173	5.819	5.819e-03	5.579	0.767
2000	7164	119.060	20166	11.300	11.607	5.804e-03	5.565	0.384

In Table 1 we examine how properties of the model vary with the number of super lobules. First, we note that as the number of super lobules increases so does the time to run the space colonization algorithm. We observe that the time to generate the 2000 SL model is roughly twice the time to generate the 5 SL model. There are several factors which are significantly more influential to the amount of time space colonization takes; namely the density of the attractor point cloud, and the space colonization parameters *D*, $d_k$, and $d_i$. In this convergence study, these parameters are fixed, but in general, for a less detailed model (e.g. 5, 10, 25, 50 SLs), a less dense point cloud is permissible, leading to significant improvements in runtime. This is because at every iteration of the space colonization algorithm, we perform distance computations between all existing nodes in the model and all remaining attractors in the point cloud. With the point clouds used in this paper, we cannot consistently generate models with more than 2000 SLs. It is possible to sample more dense point clouds and generate models with more super lobules, but this would incur added computational cost due to more attractor distance computations, as well as more iterations of the algorithm. This would also impact our ability to quickly query the model for varying boundary conditions. Similarly, for smaller space colonization parameters *D*, $d_k$ and $d_i$, it will take more iterations to create the model, and more node-attractor distance computations will be performed.

Next, we observe the size of the matrix which is solved to compute the pressures and flows of the model for our simulations. This matrix is of size ${\mathbb {R}}^{n\times n}$ where *n*, as previously defined is the total number of nodes, vessels and super lobules in the model. Currently, this matrix is implemented as a dense matrix, leading to significant increases in computational cost when solving the linear system for models with large *n*. The matrix is solved repeatedly when using the bisection method to find the $R_{SL}$, with the average time to solve the linear system once shown as “Time Matrix Solve”.

This brings us to our next point: how the super lobule resistance $R_{SL}$ varies with the number of super lobules in our model. Perhaps counterintuitively, as the number of super lobules in the model increases, the resistance assigned to each individual super lobule increases. However, when we consider the super lobules are arranged in a parallel manner, we can compute the effective equivalent resistance of the super lobules in the model as $1/R_{SL,eq} = nSL/R_{SL}$. From $R_{SL,eq}$ we see that all models have roughly the same equivalent resistance contributed by the super lobules, with $R_{SL,eq}$ decreasing slightly in models with more super lobules. Between the resistance contributed by the explicitly modeled vessels and the resistance assigned to the super lobules, all of the models have exactly the same total resistance. Thus, because models with many super lobules have more explicitly modeled vessels, the super lobules need to account for slightly less of the total resistance in the model.

Next, we observe the average pressure drop across each super lobule, $\Delta P_{SL}$. Similar to $R_{SL,eq}$, we observe roughly the same pressure drop for all model depths, with a slight decrease in pressure drop for larger models due to the increased vascular resistance from more explicitly modeled vessels. Last, we compute the average flow rate through the super lobules, $Q_{SL}$, for each model. The total flow rate through the models is $Q_{in} = 767$ mL/min, so the average flow rate through each super lobules should be $Q_{in}/nSL$, which is exactly the case here. Overall, we see increased computational cost in models of greater depth, as well as trends in resistance, pressure, and flow rate that are explained when we consider the structure of the models.

At this point, we do not designate a specific model as the optimal model. Instead, we highlight the ability of our approach to generate specific and highly variable models as a benefit to the end user. The user is able to trade off computational cost for additional model fidelity in low and high super lobule models, respectively. In future works where we consider heat transfer, composition of cryoprotective agents and various preservation protocols, the choice of model will become an important decision. High super lobule models will allow for higher fidelity heat transfer simulations, and more computationally efficient low super lobule models will allow for rapid exploration of the complex cryopreservation design space.

### Model variability

**Fig. 6:**
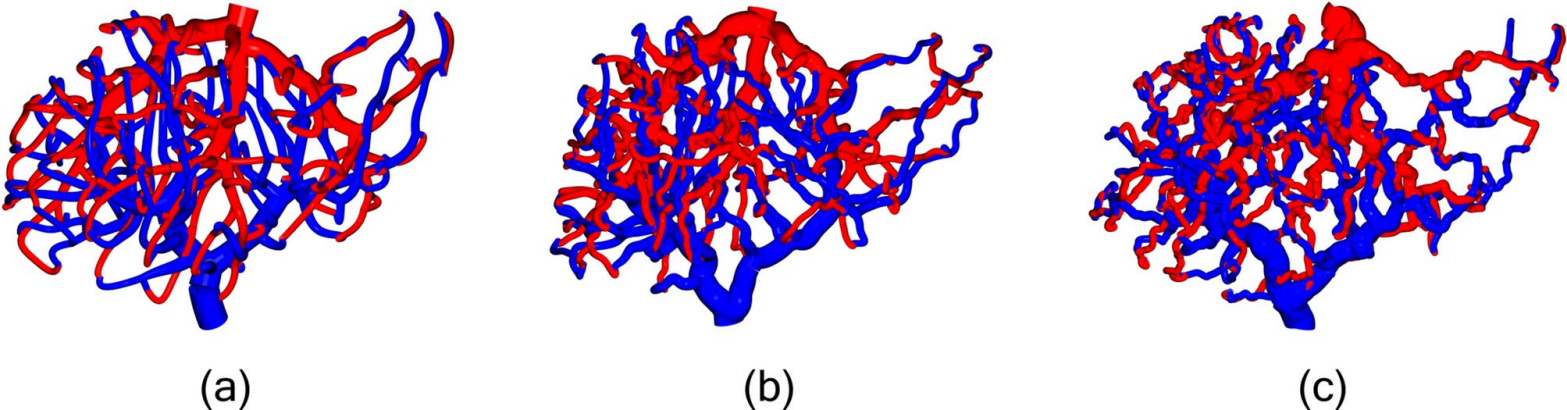
100 SL models generated with varying space colonization algorithm parameters. Larger values for *D*, $d_k,$ and $d_i$ lead to more direct models as in (**a**) where $D = 10, d_k = 15, d_i = 25$. Smaller values lead to greater tortuosity as in, as in (**c**) where $D = 2.5, d_k = 5, d_i = 10$. (**b**) corresponds to the parameters used throughout the paper where $D = 5, ~d_k = 10, ~d_i = 15$.

We can also generate models with significant topological variability. This can be done several ways; by varying the density and distribution of the attractor point cloud, and by modifying the space colonization parameters *D*, $d_k$ and $d_i$ as shown in Fig. 6. The number and distribution of attractor points that the space colonization algorithm is initialized with can lead to variability in the topology of the generated model. Models with high numbers of super lobules generally perform better with more dense point clouds. Models with few super lobules can generally be generated with sparse attractor point clouds for additional computational efficiency. In this paper we always sample the attractor point cloud randomly to ensure a roughly uniform distribution. Previous publications using the CCO method have explored using different sampling distributions to generate varying topologies^17^. Due to the two stage approach we use in constructing these models: first initializing the primary branches, then growing the entire model to the super lobules, there is additional opportunity to use different point clouds and space colonization parameters for the initialization stage versus the main growth stage. This approach affords us the flexibility to generate models with specific structures as well as a broad set of highly unique models.

### Time step convergence study

**Fig. 7:**
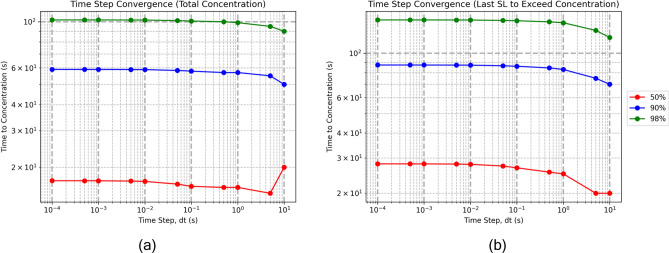
Convergence study of time step *dt* on the time to reach 50%, 90% and 98% total concentration (**a**) and for the last SL to reach those concentrations individually (**b**). Convergence plots should be read from right to left, moving from large to small time steps.

Since we simulate the loading of the vasculature in an iterative manner, it is important to determine the time step, *dt*, which provides convergence of the the loading of the vasculature. To find the time step which provides steady convergence, we look at the convergence of the concentration of the vessels and super lobules a 100 SL model. We plot the time for the total concentration of the model (all vessels and super lobules) to reach a concentration of 50%, 90% and 98%. We run the simulation for models ranging from $dt = \{0.0001, 0.0005, 0.001,\ldots , 1, 5, 10\}$ seconds. From Fig. 7 we observe that in both the total concentration and last SL cases, the time to all three concentrations has converged at a time step of $dt = 0.01~\text {seconds}$. For the remainder of simulations in this paper we use a time step of $dt = 0.01~\text {seconds}$.

### Model evaluation

**Fig. 8:**
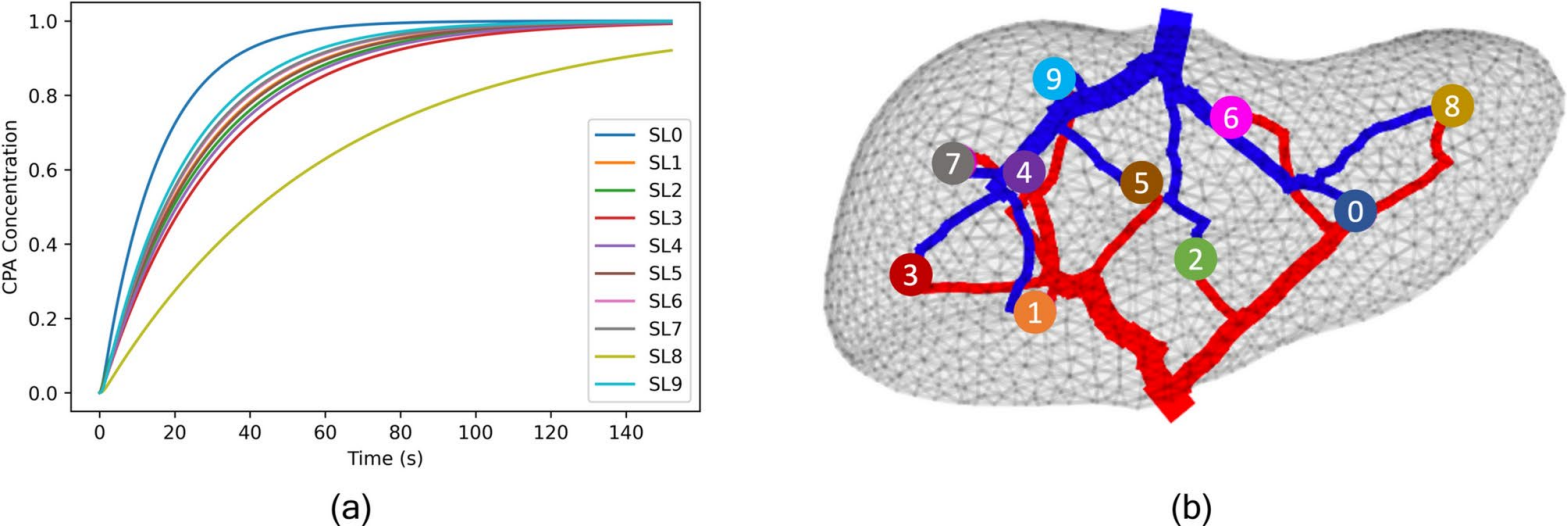
Concentration of CPA versus time in each super lobule of a 10 SL model (**a**). We note that super lobules fill at varying rates, with SL8 filling at the slowest rate. SL8 corresponds to the super lobule in the left lobe of the liver as detailed in (**b**), or Couinaud segments II and III.

Using the computed flow rates and vessel volumes, we can simulate CPA loading of the model at a steady flow rate, using experimental parameters from^32^. By tracking the concentration of CPA in all vessels and super lobules in the model we can examine how different regions of the liver fill at different rates as shown in Fig. 8. From this visualization we can identify regions of the liver that are the most challenging to load with CPA. Consistently across all models, regardless of the number of super lobules and topology, the upper right portion of the liver, corresponding to Couinaud segments II and III, is the most challenging to perfuse. This rightmost super lobule corresponds to the lowest curve in Fig. 8a and SL8 in Fig. 8b. This observation qualitatively agrees with experimental observations from computed tomography perfusion which found that Couinaud segments II and III had a statistically lower amount of portal venous perfusion compared to the rest of the liver across 50 patients with healthy livers^34^.

### Varying boundary conditions

Until this point, we have kept the pressure and flow rate boundary conditions constant across models. In the context of cryopreservation, the pressure and flow rate at which a liver is loaded or perfused are important parameters. In this section, we vary the pressure and flow rate boundary conditions for a single 100 SL model with $D = 5~{\text {mm}}$, $d_k = 10~{\text {mm}}$, and $d_i = 15~{\text {mm}}$ and examine how the boundary conditions affect the super lobule resistance, wall shear stress (WSS), and loading through the vessels of the model. We vary the pressure and flow rate boundary conditions exhaustively between $\Delta P \in [3, 12]$ mmHg in 1 mmHg increments and $Q \in [100, 1000]$ mL/min in 100 mL/min increments.

**Fig. 9:**
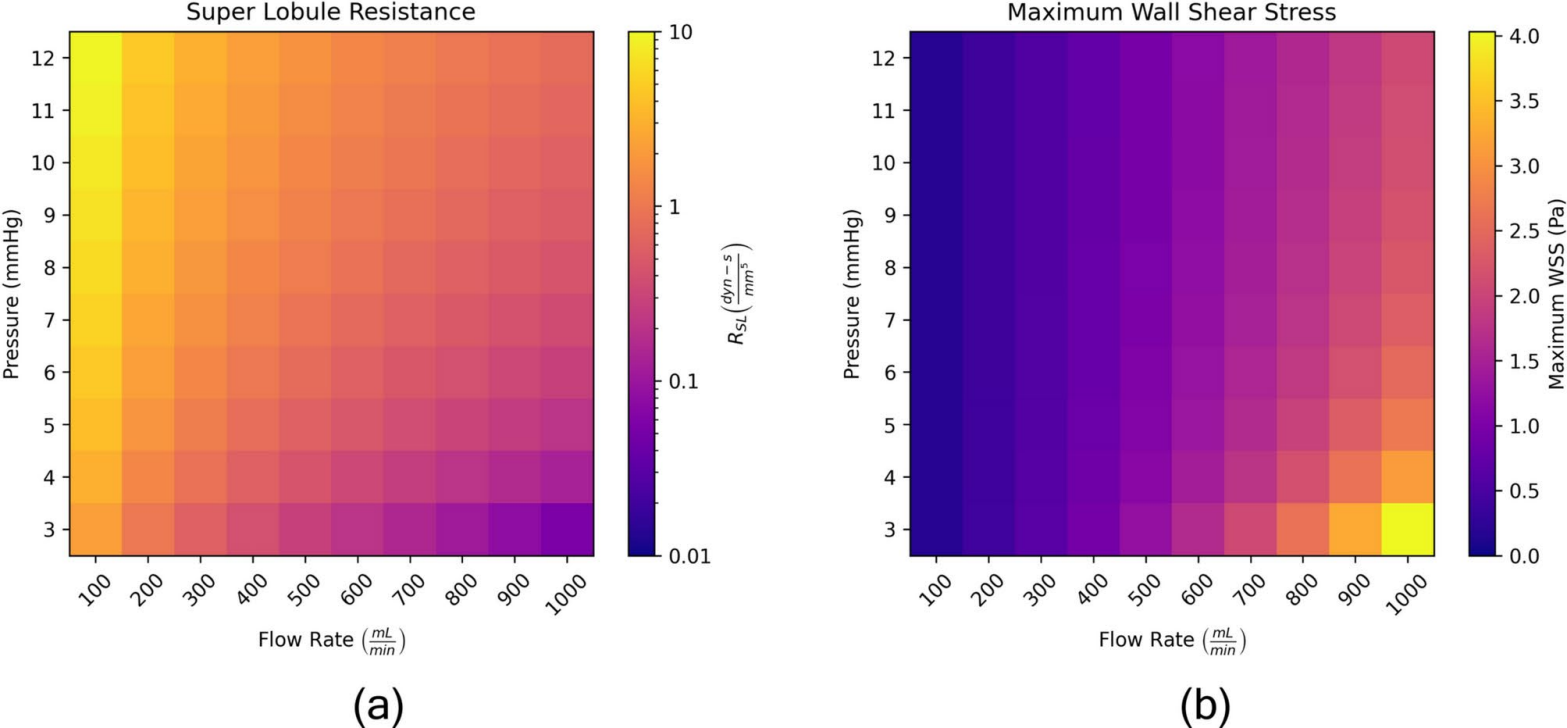
Investigating the effect of pressure and flow rate boundary conditions on the superlobule resistance (**a**), maximum wall shear stress (**b**) for a 100 SL model.

First, we examine how the super lobule resistance, $R_{SL}$, varies with the pressure and flow rate boundary conditions. Recall that when solving the linear system of equations, we first prescribe the pressure across the system, then solve for the $R_{SL}$ which permits the prescribed bulk flow rate through the system. This means that for the same exact topological model, 100 SL in this case, the super lobule resistance will vary based on the prescribed pressure and flow rates. There also exist cases where the equivalent resistance of the modeled vessels is higher than the equivalent resistance required by the pressure and flow rate prescribed to the system. More precisely, there does not exist a non-negative $R_{SL}$ that would permit the prescribed flow rate through the system. For the provided 100 SL model, $\Delta P = 2$ mmHg and $Q = 1000$ mL/min is one of the aforementioned cases, hence our sweep starting at $\Delta P = 3$ mmHg. Figure 9a plots the super lobule resistance across the range of pressure and flow rate boundary conditions. For a fixed pressure, we observe as the flow rate increases, the super lobule resistance decreases. For a fixed flow rate we observe as the pressure increases, the super lobule resistance also increases. Both of these trends intuitively make sense when we consider the pressure, flow, resistance equation relation for our model is simply $\Delta P = QR$.

In Fig. 9b, we examine the maximum wall shear stress (WSS) in the model across all pressure and flow boundary conditions. While it may be desirable to perfuse the liver at a higher flow rate to achieve faster perfusion, we also must be mindful that we do not damage the blood vessels with high shear stresses. We compute the wall shear stress, $\tau$, in a vessel according to the vessel flow rate, *Q*, fluid viscosity, $\mu$, and vessel radius, *r*, as detailed in Eq. 12.

12 $$\begin{aligned} \tau = \frac{{4} \mu Q}{\pi r^3} \end{aligned}$$

**Fig. 10:**
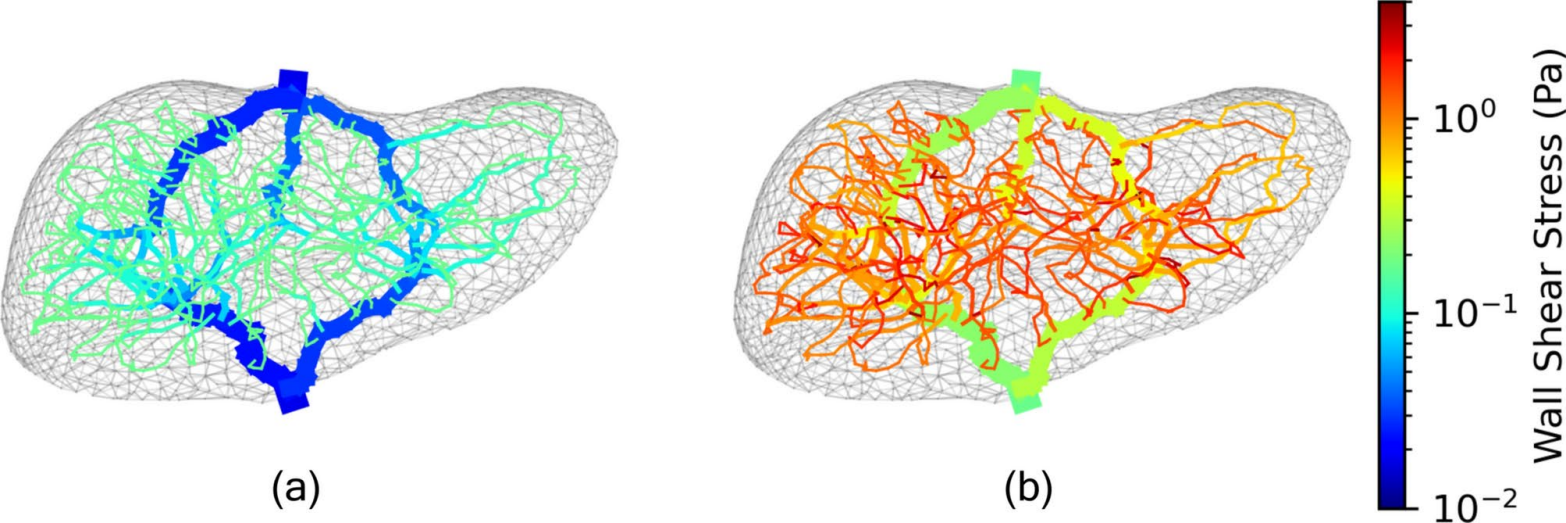
Wall shear stress throughout the vessels in the case (P = 12 mmHg, Q = 100 mL/min) with the lowest maximum WSS (**a**) and the case (P = 3 mmHg, Q = 1000 mL/min) with the highest maximum WSS (**b**).

Across all models, we note that the WSS is highest in the smaller scale vessels, which is consistent with results from Tithof et al.^13^. These small vessels are also the most fragile, with thin walls that are easily damaged by high shear stresses. Smaller radii lead to high shear stresses due to the inverse cubic relation of WSS to internal radius term in Eq. 12. As we would expect, higher flow rates lead to higher wall shear stresses. Perhaps counter-intuitively, lower pressures lead to a higher maximum wall shear stress across a given flow rate. This is because for a given flow rate, a lower pressure model has a lower super lobule resistance as evidenced in Fig. 9a. Models with high super lobule resistances lead to more even distribution of flow at the super lobule level, and conversely models with low super lobule resistance are more likely to have vessels with relatively high flow rates that lead to higher maximum wall shear stresses. This phenomenon is further explained in the discussion section. We visualize the WSS in all vessels for the highest and lowest maximum WSS cases in Fig. 10. Again, this type of analysis would be very useful when deciding which boundary conditions to utilize when designing a cryopreservation protocol.

**Fig. 11:**
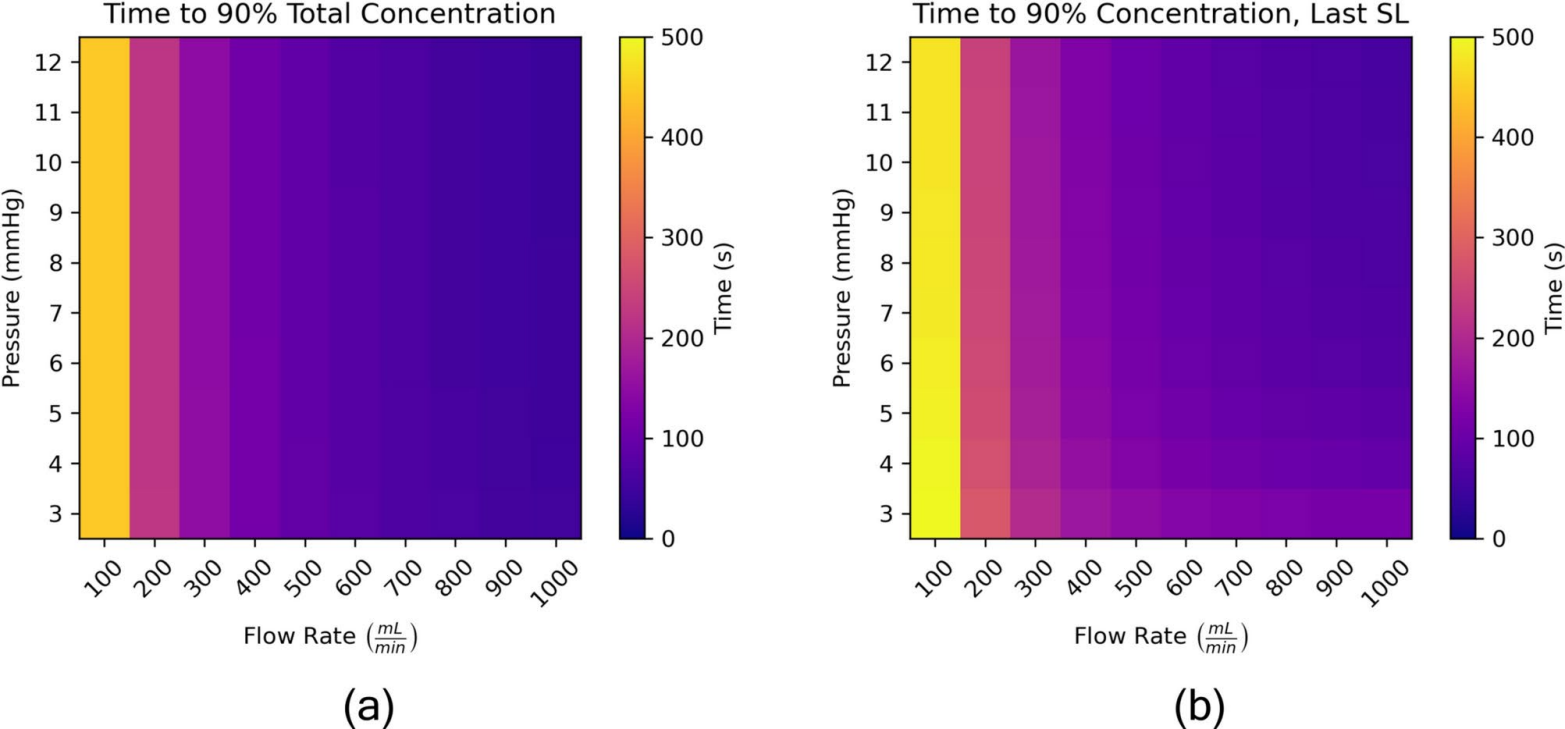
Investigating the effect of pressure and flow rate boundary conditions time to load to 90% CPA concentration for the entire model (**a**) and the slowest to fill super lobule (**b**) for a 100 SL model.

**Fig. 12:**
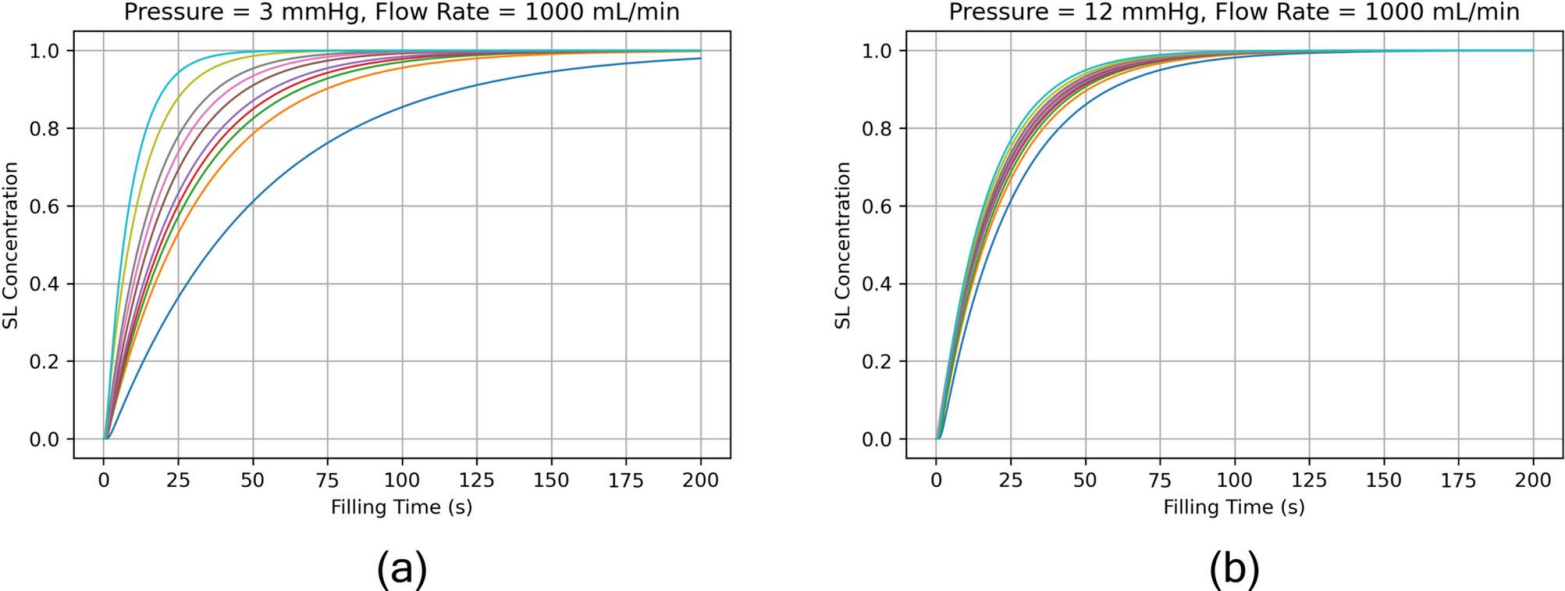
Super lobule concentration versus time in for two cases in Fig. 11. Curves for only 10 of the 100 SLs are plotted. SLs are sorted by concentration rank order and 10 evenly spaced SLs are chosen, including the slowest and fastest to fill SLs.

In Fig. 11 we examine how models and super lobules fill over time for the varying pressure and flow boundary conditions. We consider two cases: when the total concentration of CPA in the model exceeds 90%, and when the concentration of all super lobules in the model exceeds 90%. We refer to the latter case as the “last SL” case. The last super lobule case is most interesting to us, as we are trying to identify regions of the liver which are most difficult to load with CPA. We observe in both the total concentration and last super lobule cases, as flow rate increases so does the rate of loading. In the total concentration case, the pressure has no effect on the rate at which the model fills, since this is simply a product of the inlet flow rate. More interestingly, we observe in Fig. 11b that as pressure increases, there is a slight reduction in the time it takes for the last super lobule to reach 90% concentration. Again, this relates to the fact that for higher pressures, there is higher super lobule resistance, which has the effect of creating more uniform flow across all super lobules. We can see this phenomenon more clearly in Fig. 12, where we load both models at a fixed flow rate of $Q = 1000$ mL/min, and Fig. 10a has a pressure of 3 mmHg, while Fig. 10b has a pressure of 12 mmHg. We note that the low pressure model has a high amount of disparity in the rates at which its super lobules load, while the high pressure model sees a much tighter dispersion of loading rates. Figure 12 also can help support the notion for a fixed flow rate, low pressure, and therefore low $R_{SL}$ models, there are higher maximum wall shear stresses due to the fact that the super lobules themselves experience greater variance in flow rates. When the pressure is increased, the super lobule resistance increases and serves to even out the flow rates across all super lobules.

## Discussion

In this work we adapt the space colonization algorithm to generate models of the liver vasculature. We show that this method is able to generate models of varying depth and topology which adhere to bulk flow conditions thanks to the introduction of the novel super lobule concept. With an electrical circuit analogy the flows and pressures in the models can be quickly solved, and the perfusion of the models qualitatively agree with experimental observations from real human livers. This approach can be used to generate and simulate large numbers of self similar or widely variable models. We illustrate the ability of the approach to investigate the loading of models for varying pressure and flow rate boundary conditions, and examine the effects associated with varying these boundary conditions.

With our broader goals of simulating perfusion and heat transfer throughout the liver in mind, it was important that our model was both three-dimensional and fully connected such that we could simulate flow through the vasculature and retain spatial relationships between the vessels. The fully connected requirement led us to not build on previous three-dimensional algorithms such as CCO^14,16–18,35^, or three-dimensional anatomically generated models^10^. There do exist models which fully connect the inlet and outlet networks, but these models do not meet our other primary requirement: three-dimensional information must be retained^11,13,36^. Tithof et al. and Wang et al. do retain some spatial relationships in their model, with regions corresponding to the Couinaud segmentation, however to perform heat transfer simulations while loading the model, we will require a complete three-dimensional representation^13,36^. Therefore, we built upon the space colonization algorithm which generates the three-dimensional vasculature in an iterative manner similar to CCO, but provides us a natural mechanism to connect the model.

When making any model, modeling assumptions must be carefully considered. Perhaps the biggest simplification we made was to not model the hepatic artery. We noted that the portal vein is responsible for the majority of the blood supply to the liver and the hepatic artery follows a trajectory similar to the portal vein. That said, the hepatic artery also operates at largely different pressures and flow rates, and it would be interesting to analyze the behavior of our model with both the portal vein and the hepatic artery modeled. There are some perfusion techniques which only perfuse the portal vein^27^, but there is debate whether this approach is sufficient^37–39^. Currently, most modern perfusion and preservation methods perfuse both portal vein and hepatic artery. As we build upon this model in future works, we intend to include the hepatic artery, which can be generated and connected with the space colonization algorithm, just as we generated the portal and hepatic veins.

**Fig. 13:**
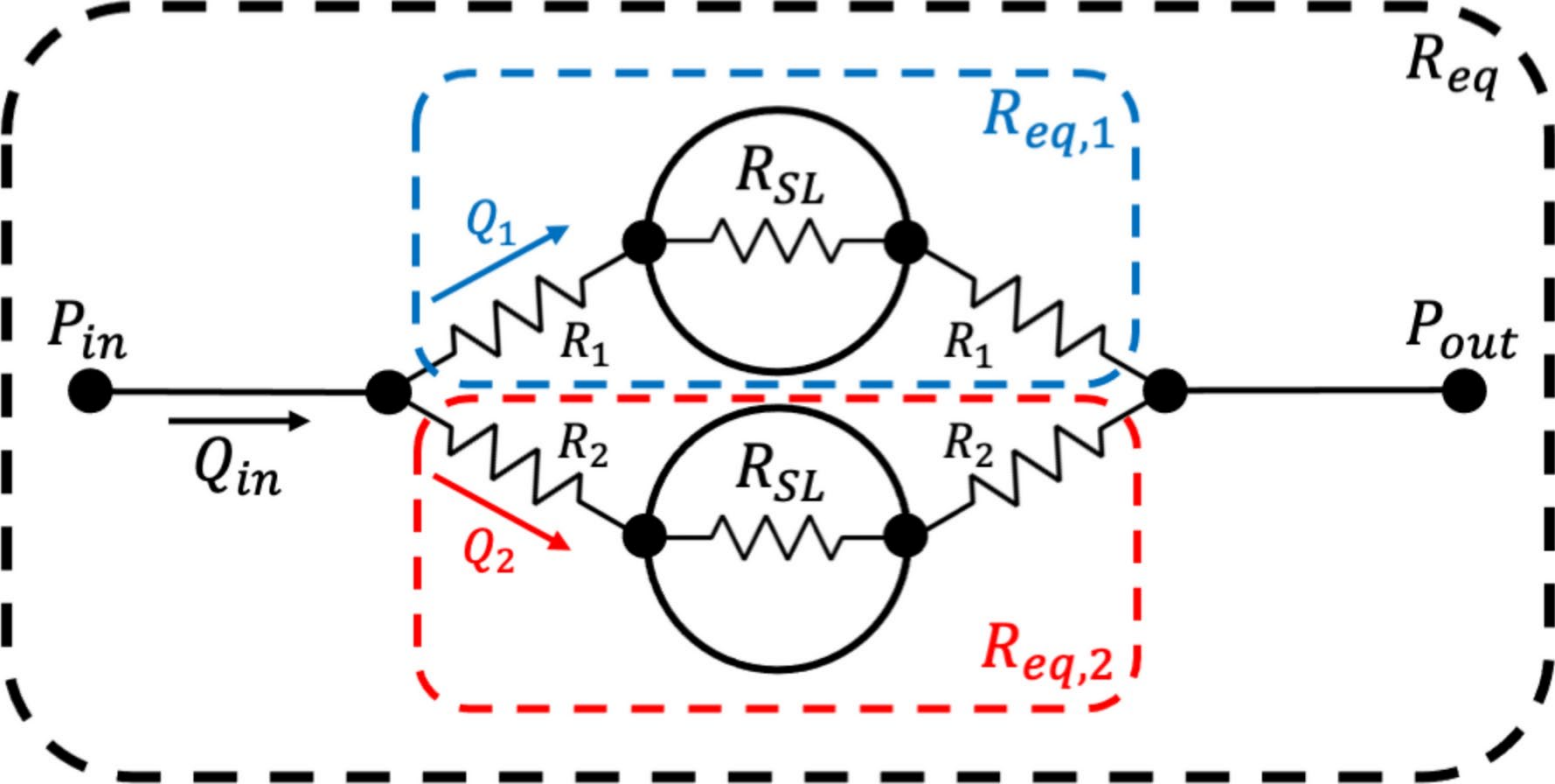
Simple super lobule resistance network used for calculations in Table 2.

With the introduction of the super lobule, we make additional assumptions. We assign a lumped resistance to all of the super lobules in our model, to represent the small scale vessels which we do not explicitly model. In the results section, we highlight that the super lobule resistance is dependent on both pressure and flow rate boundary conditions prescribed to the model. In this way, we can generate models of varying depth and topology that adhere to the same bulk flow conditions by solving for the correct super lobule resistance. However, it may not be physiologically accurate to allow our models to vary the super lobule resistance so significantly, especially considering the effect this can have on the distribution of flow throughout the model. This effect is seen in maximum wall shear stress (Fig. 9b), the time to load the last super lobule (Fig. 11b), as well as the super lobule concentration over time (Fig. 12). To further explain this phenomenon, we present a simplified example in Fig. 13. Here we set specific resistances to the vessels in our model $\left( R_1, R_2\right)$, as well as the super lobules $\left( R_{SL}\right)$, and we impose a flow rate, $Q_{in}$. We then solve for the equivalent resistance of the network, $R_{eq}$, the sub-networks $R_{eq,1}$ and $R_{eq,2}$, and the pressure drop across the model $\Delta P$. From this, we can compute the flow rates across each super lobule, $Q_1$ and $Q_2$. As shown in Table 2 we observe that in the symmetric case, the value of $R_{SL}$ has no effect on the distribution of flow between $Q_1$ and $Q_2$. However, for asymmetric networks, high values of $R_{SL}$ will dominate the equivalent resistance of the sub-networks and even out the flow across the super lobules. This precisely explains why for a fixed flow rate, high pressure models, therefore high $R_{SL}$ we see a tighter dispersion of rates at which the super lobules load, as well as lower maximum wall shear stresses compared to their low pressure, low $R_{SL}$ counterparts. This behavior is a byproduct of using the super lobule resistance as a free parameter, and is likely not physiologically accurate. In future works, we intend to further investigate methods to scale the resistances at the super lobules, or throughout all vessels such that the flow is more consistent across identical models and with physiological flows.

**Table 2:** Varied resistance values result in no change in flow for symmetric networks, but heavily influence the distribution of flow in asymmetric networks for the simple resistance network in Fig. 13.

	Imposed				Calculated					
	$Q_{in}$	$R_1$	$R_2$	$R_{SL}$	$R_{eq,1}$	$R_{eq,2}$	$R_{eq}$	$\Delta P$	$Q_1$	$Q_2$
Symmetric	1	1	1	0	2	2	1	1	**0.5**	**0.5**
	1	1	1	100	102	102	51	51	**0.5**	**0.5**
Asymmetric	1	1	10	0	2	20	1.82	1.82	**0.91**	**0.09**
	1	1	10	100	102	120	55.14	55.14	**0.54**	**0.46**

As we build on these models in future works, it worthwhile to revisit some of the other modeling assumptions we have made. First, we perform the simulated loading of our models with an Eulerian approach, where we require a sufficiently small time step to get converged results. As we move forward to include heat transfer in these simulations, we will need to either iteratively step between the flow and heat transfer updates, or we could represent the system with coupled ODEs that could provide much more accurate results.

Another area for improvement in the model is to account for the vasoconstriction and vasodilation of the liver vasculature. This could be achieved by including capacitances for each of the vessels in our resistance network. When livers are first perfused, the vessels can be vasoconstricted and are highly resistive to flow. Over the course of perfusion or loading, these vessels can dilate leading to a change in resistance. Similarly, over the course of a cryopreservation protocol, the change in temperature has a large influence on the constriction and dilation of the vessels^40^. If we were to modify our resistance network to include capacitances, we would need to solve a system of differential equations rather than a system of linear equations. The change in temperature also has a considerable impact on the viscosity of the CPAs which are flowing throughout the system, which could be updated over the course of our simulations. Lastly, as we consider the impact of viscosity on the model flows, it is important to recognize that the Hagen-Poiseuille model does not account for changes in viscosity of non-Newtonian fluids due to shear forces. While the Hagen-Poiseuille model is commonly used in models of vascular flow, it could be important to account for the affect of shear forces especially in regions of high flow or high WSS by including more complex fluid models in our system.

Overall, we view this work as a first step towards building models which can assist in the development of complex cryopreservation protocols. While we will never be able to capture the true complexity of biological systems, computational models can contribute significantly to the exploration of large design spaces, especially considering the limited access to scarce donor organs such as the human liver. In future works we intend to focus on optimizing and improving our models to more closely reflect the physiology of the real human liver, while adding complexity to the simulations that allow us to simulate loading of the vasculature across varying temperatures. It is critical that we validate future models against experimental and anatomical data to ensure that our models are clinically relevant. Then, we can truly analyze the effect of varying pressure, flow rate, and temperature boundary conditions on the perfusion and freezing of the system.

## Data Availability

The code used in this work can be found at: https://github.com/danemerson13/space-colonization-py. Many of the results files are too large to be hosted on Github and can be made available upon request to danielem@andrew.cmu.edu or lkara@cmu.edu.
